# Adding genetic scores to risk models in colorectal cancer

**DOI:** 10.18632/oncotarget.27110

**Published:** 2019-08-06

**Authors:** Carla J. Gargallo-Puyuelo, Ángel Lanas, María Asunción García-Gonzalez

**Affiliations:** Department of Gastroenterology, Hospital Clínico Universitario Lozano Blesa, Zaragoza, Spain; Aragón Health Research Institute, Zaragoza, Spain

**Keywords:** colorectal cancer, colorectal adenoma, genetic risk score, prediction risk models

Colorectal cancer (CRC) represents the second most common cancer worldwide and the third leading cause of cancer death. Most CRCs arise from premalignant colorectal lesions (mainly adenomas) that require years to develop an invasive disease. Early stage detection through the use of screening programs can sharply reduce CRC incidence and mortality allowing for better outcomes of the disease. The effectiveness of these programs may be strongly enhanced by targeting screening to individuals at higher risk.

CRC is a multifactorial disease resulting from complex interactions between environmental and genetic factors. A great progress in understanding the underlying genetic factors of CRC has been made in the past two decades. Since the first genome-wide association study (GWAS) on CRC risk published in 2007 by *Tomlinson et al*. [1], more than 80 common single nucleotide polymorphisms (SNPs) in low-penetrance genes have been identified, with the majority of GWAS been conducted among European ancestry populations. It has been found that many of these risk variants lie in loci nowhere near known coding or regulatory regions, opening new exciting doors to mechanisms and pathways not previously known. Unlike the numerous studies performed in CRC, the relevance of genetic susceptibility in the development of premalignant colorectal lesions has been less evaluated. Colonic adenomas probably represent an intermediate phenotype between asymptomatic individuals carrying risk variants and CRC. A deeper knowledge of genetic factors related to premalignant lesions risk can also provide insight into the biological and genetic mechanisms relevant to initiation and progression of CRC. A recent study by *Gargallo et al.* [2] showed that several SNPs associated with CRC risk in previous GWAS (rs10505477, rs6983267, rs10795668, and rs11255841), are also involved in the susceptibility to colorectal adenomas or specific high- and low-risk adenoma subtypes. Of interest, the authors found these associations to be modified by the presence of family history of CRC. The low penetrance of most identified genetic variants associated with CRC does not provide clinically relevant information on their own, but combination of risk-associated alleles in a polygenic model has been reported to increase the risk of CRC in an additive or exponential way.

Nowadays CRC screening guidelines are based mainly on age and family history. However, the elaboration of prediction risk models including environmental and genetic risk factors may allow a more accurate selection of low-and high-risk patients. Improving risk stratification will optimize the use of invasive technology and increase adherence to screening programs. The first risk prediction models for CRC were performed based on family history, lifestyle factors, and environmental risk factors. However, recent studies showed an increasing interest in developing genetic risk scores (GRS), combining common genetic variants associated with CRC for a more personalised risk assessment. In this context, *Dunlop et al*. [3], *Yarnall et al.* [4], and *Jung et al.* [5] developed prediction models that accounted lifestyle, environmental, and genetic factors, reporting a discriminatory accuracy using the area under the curve (AUC) with values ranging from 0.59 to 0.74. *Hsu et al.* [6] developed sex- and site-specific models based on family history data and 27 SNPs adjusted for endoscopy history*.* They observed that adding the GRS to prediction models increased discriminatory accuracy from 0.51 to 0.59 (*P* = 0.0028) in men and from 0.52 to 0.56 (*P* = 0.14) in women, compared to risk models based only on family history. Subsequent studies show similar results. *Ibáñez-Sanz et* al. reported a discriminatory accuracy value of 0.63 for CRC risk prediction model combining some modifiable risk factors (alcohol, obesity, physical activity, red meat and vegetable consumption, and nonsteroidal anti-inflammatory drug use), family history of CRC, and a GRS based on 21 susceptibility SNPs [7]. More recently, *Weigl et al.* [8] derived a GRS based only in a significant number of common variants (48 SNPs) previously associated with CRC. They observed that participants in the upper tertile of the GRS had a 2.7-fold increase in risk of advanced neoplasms (advanced adenomas and CRC) compared to those in the lower tertile. An increasing number of SNPs associated with CRC risk (63 SNPs, G-score) was evaluated by *Jeon et al.* [9] along with family history data and 19 life-style and environmental factors (E-score). The model combining all scores estimated CRC risk with a discriminatory accuracy value of 0.63 for men and 0.62 for women, higher in both genders when comparing to those models based only in family history or E-score and G-score separately. In line with these results, *Balavarca et al.* [10] after evaluate environmental factors and GRS for advanced colorectal neoplasm, they reported higher prediction values (AUC = 0.63) in the combined environmental-genetic score model compared with single environmental score (AUC = 0.584, *p* = 0.0002).

All these studies support the idea that adding genetic, environmental and lifestyle information into a CRC risk prediction model may significantly increase the discriminatory accuracy over models using only age and family history. Risk stratification could still be improved by integrating new discovered susceptibility SNPs to GRS as well as other relevant biomarkers such as epigenetic markers. Combining environmental, lifestyle factors and GRS in risk prediction models can help to tailor CRC prevention measures by adapting the onset age, nature and the intensity of CRC screening strategies.
